# Effects of cognitive reserve proxies on cognitive function and frontoparietal control network in subjects with white matter hyperintensities: A cross‐sectional functional magnetic resonance imaging study

**DOI:** 10.1111/cns.13824

**Published:** 2022-03-11

**Authors:** Qing Ye, Huahong Zhu, Huiping Chen, Renyuan Liu, Lili Huang, Haifeng Chen, Yue Cheng, Ruomeng Qin, Pengfei Shao, Hengheng Xu, Junyi Ma, Yun Xu

**Affiliations:** ^1^ Department of Neurology Drum Tower Hospital Medical School of Nanjing University Nanjing China; ^2^ The State Key Laboratory of Pharmaceutical Biotechnology Institute of Brain Science Nanjing University Nanjing China; ^3^ Jiangsu Province Stroke Center for Diagnosis and Therapy Nanjing China; ^4^ Nanjing Neuropsychiatry Clinic Medical Center Nanjing China; ^5^ Department of Neurology Nanjing Drum Tower Hospital Clinical College of Nanjing Medical University Nanjing China; ^6^ Department of Radiology Drum Tower Hospital Medical School of Nanjing University Nanjing China

**Keywords:** cognitive reserve, education, leisure time activity, white matter hyperintensities, working activity

## Abstract

**Aims:**

This study aimed to analyze the potential association between cognition reserve (CR) components, including education, working activity, and leisure time activity, and cognitive function in subjects with white matter hyperintensities (WMH). The study also explored the role of the frontoparietal control network (FPCN) in such association.

**Methods:**

White matter hyperintensities subjects with and without cognitive impairment (CI) were evaluated with multimodal magnetic resonance imaging, neuropsychological testing, and CR survey. FPCN patterns were assessed with dorsolateral prefrontal cortex seed‐based functional connectivity analysis.

**Results:**

Education was positively associated with cognitive function in WMH subjects with or without CI, whereas working activity and leisure time activity were positively associated with cognitive function only in those without CI. Similarly, education was associated with bilateral FPCN in both WMH groups, whereas working activity and leisure time activity were associated with bilateral FPCN mainly in the group without CI. Furthermore, FPCN partially mediated the association between education and cognitive function in both WMH groups.

**Conclusion:**

Education showed a positive impact on cognitive function in WMH subjects regardless of their cognitive status, whereas working activity and leisure time activity exhibited beneficial effects only in those without CI. The FPCN mediated the beneficial effect of education on cognitive function.

## Graphical abstract

Education regulated cognitive function both before and after the onset of cognitive impairment in subjects with white matter hyperintensities, whereas working activity and leisure time activity displayed effects mainly before the onset of cognitive impairment.

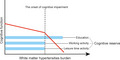

## 1 INTRODUCTION

White matter hyperintensities (WMH), the most common morphologic feature on brain magnetic resonance imaging (MRI) in cerebral small vessel disease, is common among older people.^1^, ^2^, ^3^ There is increasing evidence suggesting that WMH can cause cognitive decline and plays a significant role in the etiology of vascular cognitive impairment (CI).^4^, ^5^ However, individuals with WMH exhibit high levels of heterogeneity in cognitive performance, and a portion of these individuals even maintain normal cognitive function.^6^, ^7^ This high heterogeneity may be related to the effect of cognitive reserve (CR) activities.

Cognitive reserve is a theoretical concept that explains the individual differences in maintaining cognitive function in the face of brain pathology. First, greater CR appears to be associated with better cognitive performance in WMH subjects.^6^, ^8^, ^9^ Second, CR may act as a moderator between the burden of WMH and cognitive performance. A more significant burden of WMH links to poorer cognitive performance in subjects with low CR. In contrast, this association tends to be weakened or disappear in subjects with high CR.^10^, ^11^, ^12^ CR is commonly measured with proxies, and the Cognitive Reserve Index questionnaire (CRIq) evaluates CR from three aspects: education, working activity, and leisure time activity.^13^ The three aspects contribute independently and differentially to CR.^14^ Whether these proxies play distinct roles in affecting cognitive function in WMH subjects remains elusive.

Recent studies on Alzheimer's disease (AD) have revealed stage‐dependent effects of CR on cognitive function across the AD spectrum. CR's positive effects on cognition are stronger in predementia stages than in dementia stages.^15^, ^16^ High CR attenuates the cognitive decline in predementia stages, but accelerates cognitive decline in dementia stages.^17^ For WMH subjects, Zahodne et al.^18^ showed that education mitigated the effect of WMH on cognitive function in subjects at lower risk for dementia, but exacerbated the effect in those at higher risk for dementia. To the best of our knowledge, whether the effects of CR on cognition differ between WMH subjects with or without CI remains unclear.

The link between CR and brain activities has been widely explored using task‐based functional magnetic resonance imaging (fMRI) techniques. Colangeli et al.^19^ performed a meta‐analysis of 17 fMRI studies and found that higher CR was associated with greater activation in frontal, parietal, and anterior cingulate regions in healthy elderly subjects. The frontoparietal control network (FPCN) is thought to flexibly support multiple resting‐state networks, e.g., the default mode network and dorsal attention network, to complete cognitive tasks, thus serving as a “regulating” role.^20^, ^21^ Alterations in FPCN patterns are related to executive function and attention, commonly affected in vascular CI.^22^, ^23^, ^24^ Our recent resting‐state fMRI study found that an altered FPCN pattern, i.e., increased within‐network functional connectivity (FC) of the FPCN and decreased FC between the FPCN and the default mode network, was related to CI in subjects with WMH.^25^ However, the role of CR in such a relationship remains to be defined.

In this study, WMH subjects without CI, WMH subjects with CI, and healthy control (HC) subjects underwent multimodal MRI scans, neuropsychological testing, and CR assessment. We aimed to (1) determine the association of each CR aspect with cognitive function across the three groups and (2) explore the role of the FPCN in the association between CR and cognitive function in WMH subjects. We hypothesized that the FPCN could mediate a positive effect of CR on cognitive function in WMH subjects.

## 2 METHODS

### 2.1 Participants

This cross‐sectional study initially enrolled 144 subjects with WMH and 101 HC subjects, all of whom were recruited for the Study on Register and the Diagnosis, Therapy and Prognosis of Cerebral Small Vessel Disease (Registration number: ChiCTR‐OOC‐17,010,562) in the Drum Tower Hospital, Medical School of Nanjing University from December 2016 to May 2020. The study was carried out in accordance with the latest version of the Helsinki Declaration of 1975 and approved by the Drum Tower Hospital Research Ethics Committee. All subjects provided written informed consent.

The inclusion criteria and exclusion criteria were previously described.^26^ The inclusion criteria for subjects with WMH were as follows: (1) age >50 years; and (2) presence of WMH on brain MRI (Fazekas grade 1–3), no recent subcortical infarction, and no cerebral microbleeds. An HC group included participants showing normal global cognitive function [Montreal Cognitive Assessment (MoCA)], no presence of WMH on MRI (Fazekas grade 0), and no other MRI presentative characteristics of cerebral small vessel disease. The study excluded individuals with neurodegenerative diseases, leukoencephalopathy of presumed nonvascular origin, history of ischemic stroke (diameter of infarct >15 mm) or cardiogenic cerebral infarction, severe neurological diseases, or severe systemic diseases. Detailed exclusion criteria are shown in the Supplemental Material. Seven WMH subjects and six HC subjects were excluded due to incomplete data. Finally, a total of 137 WMH subjects and 95 HC subjects were included in the present study. The sample size may be large enough for a fMRI study.^27^, ^28^

### 2.2 Assessment of cognitive function and CR

All subjects completed a battery of neuropsychological examinations at entry. The MoCA and the Mini Mental State Examination (MMSE) were used to assess global cognitive function. The WMH subjects with MoCA scores lower than education‐adjusted norms^29^ (the cutoff was ≤19 for 1–6 years of education, ≤24 for 7–12 years of education, and <26 for >12 years of education) were classified into a WMH with CI group (*n* = 77), and other patients were assigned to a WMH without CI group (*n* = 60). The assessment of each cognitive domain is shown in the Supplemental Material. The individual scores for each domain were obtained by averaging the Z scores of the relevant neuropsychological tests. The Hamilton anxiety scale and the Hamilton depression scale were used to assess mental status.

To quantify CR, we conducted a survey with the CRIq and generated the composite index of CR, i.e., the Cognitive Reserve Index (CRI).^13^ All participants with normal cognitive function were asked questions about three indicators of CR: education, working time activity, and leisure time activity. For those with CI, the questions were asked of a family caregiver who was familiar with the present and past habits of the subject. After collecting the self‐reported information about CR, we obtained the scores for each of the three aspects of the CRIq: CRI‐education, CRI‐working activity, and CRI‐leisure time activity, as well as the total CRI for each subject. Detailed information is shown in the Supplemental Material.

### 2.3 MRI acquisition

The procedure for MRI scanning was described previously.^25^, ^30^ All subjects underwent MRI scanning on a 3.0‐Tesla MRI scanner (Ingenia 3.0T, Philips Medical Systems, Eindhoven, Netherlands) with a 32‐channel head coil. Detailed procedure is shown in the Supplemental Material.

### 2.4 Volume assessment of grey matter, whole brain, and WMH

As mentioned in our previous research,^30^ structural processing was performed using the Voxel‐based morphometry 8 (VBM8) toolbox (http://dbm.neuro.unixxjena.de/vbm8) for Statistical Parametric Mapping software (SPM12, http://www.fil.ion.ucl.ac.uk/spm). Detailed procedure is shown in the Supplemental Material. The volumes of grey matter, white matter, and cerebrospinal fluid were obtained, and the whole brain volume was calculated as the sum of these three values. Grey matter atrophy is a calculation of grey matter volume divided by the brain volume.

The volume of WMH lesions was evaluated on T1 and T2‐FLAIR images using the Lesion Segmentation Tool (LST) toolbox version 2.0.151 (http://www.statistical‐modelling.de/lst.html) for SPM12.^31^ Detailed procedure is shown in the Supplemental Material.

### 2.5 FMRI preprocessing and network mapping

The resting‐state fMRI data were preprocessed using Data Processing and Analysis of Brain Imaging (DPABI 2.3, http://rfmri.org/DPABI) software based on SPM12.^32^ Six‐millimeter radius spheres centered at the bilateral dorsolateral prefrontal cortex (DLPFC) (MNI space: −42, 34, 20/44, 36, 20) served as seed regions for the bilateral FPCN. Detailed procedure is shown in the Supplemental Material.

### 2.6 Statistical analysis

The Kolmogorov–Smirnov test was used to assess the data normality of continuous variables. Normally distributed data were presented as the mean ± standard deviation (SD) and analysed using one‐way analysis of variance (ANOVA). Non‐normally distributed data were presented as medians (interquartile range) and analysed using a Kruskal–Wallis test. Chi‐square tests were applied to compare the sex ratio among the three groups. We examined the relationship between the total CRI or each aspect of CRI (as independent variables) and the MoCA and MMSE scores (as dependent variables) using multiple linear regression analysis with adjustment for age, sex, WMH volume, whole brain volume, and grey matter atrophy rate in each group. While each aspect of CR served as an independent variable, the other two aspects of CR were additionally treated as covariates. To explore the relationship among the three aspects of CR, Pearson's correlation analyses were performed between any two aspects in each group. These analyses were performed using Statistical Package for Social Sciences (SPSS) V22.0 (IBM), and statistical significance was set at *p* < 0.05. A Bonferroni correction was performed on the results of the multiple linear regression analyses and the Pearson's correlation analyses.

In the FPCN analyses, correlative analyses in resting‐state fMRI data analysis toolkit (REST) 1.8 were performed to examine the association between the FPCN and each CR aspect while controlling for age, sex, and grey matter images. The thresholds were set at a corrected *p* < 0.05, determined by Monte Carlo simulation for multiple comparisons (voxelwise *p* < 0.05, cluster size >2646 mm^3^). The FC strength of regions with significance was extracted for further analysis. We performed Pearson's correlation analyses using SPSS to examine the detailed associations between the FC values and CR aspects. Finally, we estimated the direct and indirect (through the FC of FPCN) associations of CRI‐education with cognitive performance using a mediation analysis controlling for age and gender. Statistical significance was determined at 2‐sided *p* < 0.05, and the analyses were conducted using Hayes’ Process macro V3.5 (http://processmacro.org/index.html) in SPSS. According to the protocol,^33^ we selected Model 4 in the Process macro and computed bootstrapped (*n* = 5000) and bias‐corrected 95% confidence intervals for the mediation effects of FPCN FC.

## 3 RESULTS

### 3.1 Demographic, neuropsychological, and CR data

As shown in Table 1, no significant differences in sex, years of education, whole‐brain volume, grey matter atrophy rate, CRI, CRI‐education, or CRI‐working activity were found among the three groups. WMH groups were significantly older than the HC group. The WMH with CI group had significantly greater WMH volume, poorer performance in all cognitive domains, and lower CRI‐leisure time activity than the other two groups. Correlative analyses showed that the three aspects of CR were significantly and positively correlated in both the WMH with CI group and the HC group, not in the WMH without CI group (Table S1 in Supplemental Material).

**TABLE 1:** Demographic, neuropsychological, and CR data

Items	HC (*n* = 95)	WMH without CI (*n* = 60)	WMH with CI (*n* = 77)	*F*/Chi Square	*p*‐Value
Age, y (SD)	60.78 ± 7.36	64.78 ± 7.93^a^	65.52 ± 7.97^a^	9.853	**<0.001**
Gender (male/female)	46/49	28/32	42/35	0.998	0.732
Education, y (SD)	11.65 ± 4.75	11.37 ± 4.43	11.23 ± 3.20	0.238	0.788
Brain volume, ml (SD)	1328.6 ± 122.36	1340.74 ± 101.18	1356.38 ± 130.00	1.193	0.305
WMH volume, ml (IQR)	1.01 (0.50–2.02)	3.21 (1.49–6.67)^a^	6.10 (3.21–11.82)^a,b^	‐	**<0.001**
Grey matter atrophy, % (SD)	41.36 ± 1.85	41.04 ± 1.94	40.90 ± 1.84	1.442	0.239
MMSE (IQR)	29 (28–30)	29 (28–30)	28 (27–29)^a,b^	‐	**<0.001**
MoCA (IQR)	25 (22–27)	26 (25–27)	22 (19–23)^a,b^	‐	**<0.001**
Memory (SD)	0.1544 ± 0.8878	0.1244 ± 0.6596	−0.2215 ± 0.8081^a,b^	5.111	**0.006**
Executive function (IQR)	0.2088 (−0.4512–0.7686)	0.0245 (−0.4831–0.5499)	−0.4713 (−0.8936–0.0895)^a,b^	‐	**<0.001**
Visual‐spatial ability (IQR)	0.2841 (−0.4721–0.7883)	0.2841 (−0.4721–0.5362)	−0.2200 (−0.7242–0.2841)^a,b^	‐	**<0.001**
Processing speed (SD)	0.1929 ± 0.8896	0.1615 ± 0.8208	−0.3415 ± 0.6627^a,b^	11.65	**<0.001**
CRI (IQR)	96.00 (88.50–115.00)	95.00 (87.00–110.00)	94.00 (86.00–106.00)	‐	0.695
CRI‐education (IQR)	102.00 (93.00–112.00)	103.00 (90.00–115.50)	101.00 (94.00–112.00)	‐	0.928
CRI‐working activity (IQR)	102.00 (91.00–114.00)	104.00 (92.00–117.00)	100.00 (90.00–117.02)	‐	0.866
CRI‐leisure time activity (IQR)	87.00 (79.50–93.00)	89.00 (77.00–97.50)	84.00 (77.00–90.00)^a,b^	‐	**0.037**

Note

Values are presented as mean ± stand deviation (SD) or median (IQR, interquartile range). Grey matter atrophy is a calculation of grey matter volumes divided by the brain volume; lower values indicate more grey matter atrophy. One‐way ANOVA was applied in the analyses of age, education, brain volume, brain atrophy rate, memory, and processing speed. χ^2^ test was applied in the analysis of gender. The Kruskal–Wallis test was applied in the analyses of WMH volume, MMSE, MoCA, executive function, visual‐spatial ability, and cognitive reserve data. Significance is highlighted in bold (*p *< 0.05). ^a^ *p* < 0.05, differs from the control group. ^b^ *p* < 0.05, differs from the WMH without CI group.

Abbreviations: ANOVA, analysis of variance; CI, cognitive impairment; CRI, Cognitive Reserve Index; HC, healthy control; IQR, interquartile range; MMSE, mini mental state examination; MoCA, montreal cognitive assessment; SD, stand deviation; WMH, white matter hyperintensities.

### 3.2 Association of CR with cognitive function

As shown in Table 2, CRI and CRI‐education were positively associated with global cognitive functions (MMSE and MoCA) in all groups (except for MMSE scores in the WMH with CI group). In contrast, working activity and leisure time activity were positively associated with global cognitive functions in the WMH without CI and HC groups. After a Bonferroni correction was performed, the association of both CRI and CRI‐education with global cognitive functions remained unchanged. In contrast, CRI‐working activity and CRI‐leisure time activity were significantly associated with MoCA scores only in the HC group.

**TABLE 2:** Associations between CR and global cognitive function according to diagnosis

CR	CRI			CRI‐education			CRI‐working activity			CRI‐leisure time activity		
Cognition	HC	WMH without CI	WMH with CI	HC	WMH without CI	WMH with CI	HC	WMH without CI	WMH with CI	HC	WMH without CI	WMH with CI
MMSE	0.094^a^	0.357^a^	NS	0.083^a^	0.046^a^	NS	0.035	0.02	NS	0.073	NS	NS
MoCA	0.179^a^	0.429^a^	0.381^a^	0.191^a^	0.083^a^	0.13^a^	0.106^a^	0.044	NS	0.136^a^	0.065	NS

Note

Values depicted are unstandardized coefficients (β) from linear regression models with cognitive function as the dependent variable and CR as the independent variable, adjusted for age, sex, brain volume, WMH volume, and grey matter atrophy rate. *p* < 0.05 for all shown β values. ^a^ *p* < 0.002 (0.05/24 tests) corrected with the Bonferroni principle.

Abbreviations: CI, cognitive impairment; CR, cognitive reserve; CRI, cognitive reserve index; HC, healthy control; MMSE, mini mental state examination; MoCA, montreal cognitive assessment; NS, not significant; WMH, white matter hyperintensities.

### 3.3 Association of CR with FPCN

#### 3.3.1 FPCN mapping

Consistent with previous studies,^34^, ^35^ the FPCN encompassed the bilateral DLPFC, dorsomedial prefrontal cortex, and lateral parietal cortex (Figure S1 in Supplemental Material).

#### 3.3.2 Association of each aspect of CR with the right FPCN

**FIGURE 1:**
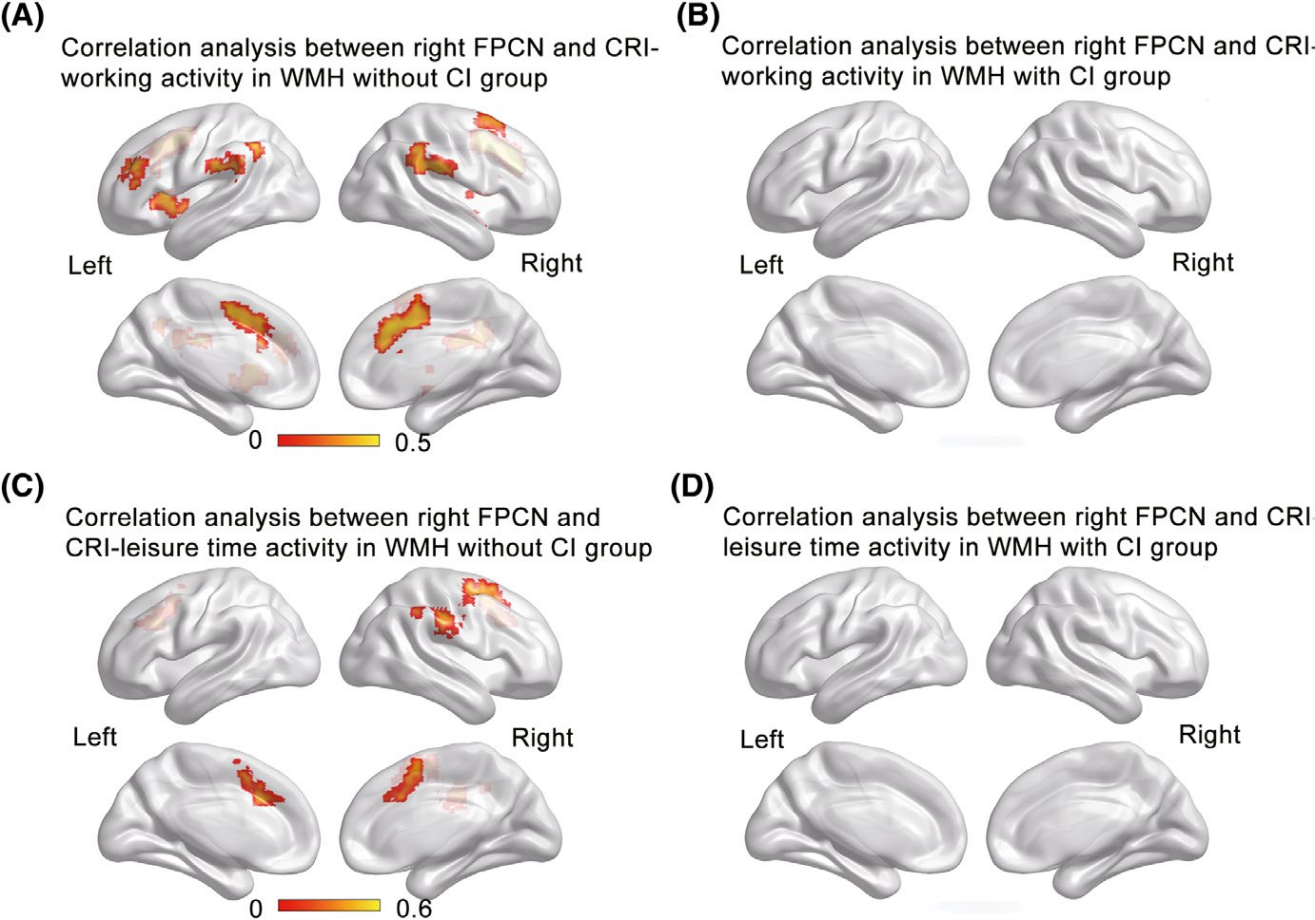
The associations of CRI‐working activity and CRI‐leisure time activity with the right FPCN in the WMH groups. (A–B) The CRI‐working activity was significantly associated with FPCN FC in the frontal, parietal, and cingulate regions in the WMH without CI group not in the WMH with CI group. (C–D) The CRI‐leisure time activity was significantly associated with FPCN FC in the frontal, parietal, and cingulate regions in the WMH without CI group not in the WMH with CI group. Correlative analyses were performed between the FPCN and each CRI aspect while controlling for age, sex, and grey matter images. The thresholds were set at a corrected *p* < 0.05, determined by Monte Carlo simulation for multiple comparisons (voxelwise *p* < 0.05, cluster size >2646 mm^3^). The color bars present correlation coefficients. CI, cognitive impairment; CRI, cognitive reserve index; FC, functional connectivity; FPCN, frontoparietal control network; WMH, white matter hyperintensities

**FIGURE 2:**
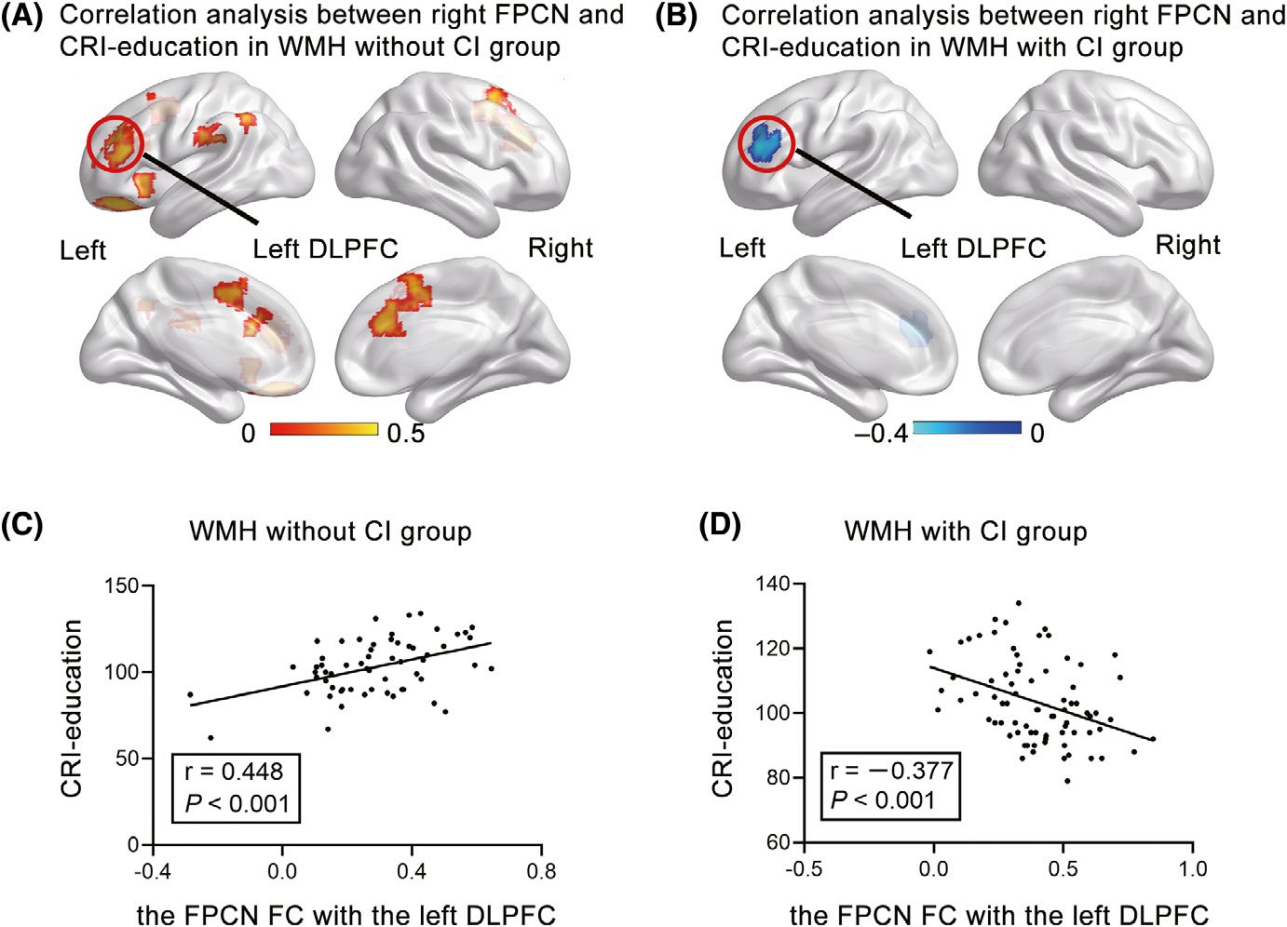
The associations of CRI‐education with the right FPCN in the WMH groups. (A–B) CRI‐education was significantly associated with FPCN FC in the left DLPFC in WMH subjects with or without CI. Correlative analyses were performed between the FPCN and each CRI aspect while controlling for age, sex, and grey matter images. The thresholds were set at a corrected *p* < 0.05, determined by Monte Carlo simulation for multiple comparisons (voxelwise *p* < 0.05, cluster size >2646 mm^3^). The color bars present with correlation coefficient. (C) FPCN FC in the left DLPFC was positively associated with CRI‐education in the WMH group without CI. (D) The FPCN FC in the left DLPFC was negatively associated with CRI‐education in the WMH group with CI. The FC values were transformed to Z scores using Fisher's Z‐transformation. CI, cognitive impairment; CRI, cognitive reserve index; DLPFC, dorsolateral prefrontal cortex; FC, functional connectivity; FPCN, frontoparietal control network; WMH, white matter hyperintensities

Both CRI‐working activity and CRI‐leisure time activity were significantly associated with the FC of the right FPCN in frontal, parietal, and cingulate regions only in the WMH without CI group (Figure 1A,C), not in the WMH with CI group (Figure 1B,D). In contrast, CRI‐education was significantly associated with the FC in the left DLPFC in both WMH groups (Figure 2A–B), although the association was significantly positive and negative in the WMH without CI group (Figure 2C, *r* = 0.448, *p *< 0.001) and the WMH with CI group (Figure 2D, *r* = −0.377, *p* < 0.001), respectively. Similarly, the FC in the left DLPFC was significantly positively and negatively associated with cognitive function in the WMH without CI group and the WMH with CI group, respectively (Figure S2 in Supplemental Material).

**FIGURE 3:**
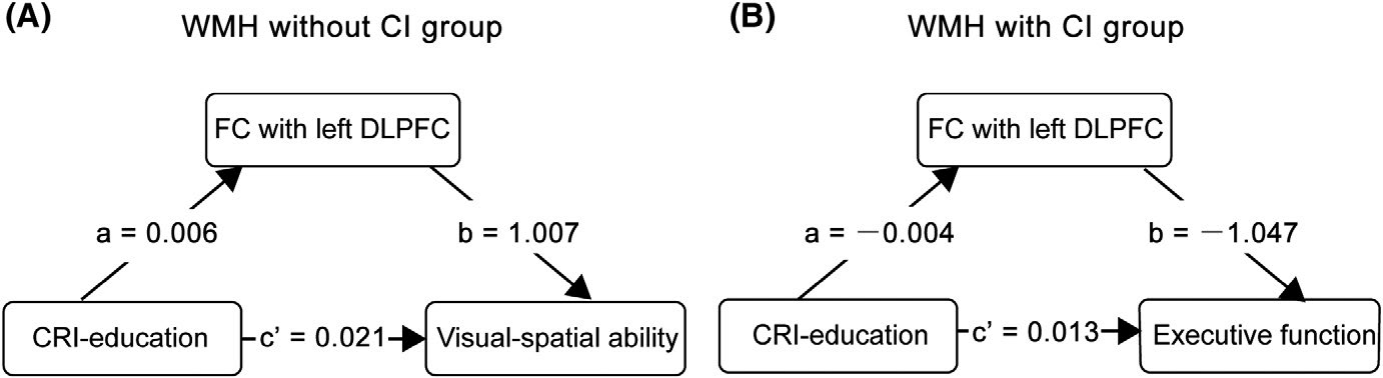
The mediating effect of the left DLPFC on the association between CRI‐education and cognitive function. (A) In the WMH group without CI, FC in the left DLPFC significantly mediated the association of CRI‐education with the visual‐spatial ability (indirect effect: 0.039; 95% CI: 0.001, 0.062). (B) In the WMH group with CI, FC significantly mediated the association of CRI‐education with executive function (indirect effect: −0.004; 95% CI: 0.001, 0.027). The associations of FC with CRI‐education and cognitive function were opposite between the two WMH groups. a, b, and c present regression coefficients. CI, cognitive impairment; CRI, cognitive reserve index; DLPFC, dorsolateral prefrontal cortex; FC, functional connectivity; FPCN, frontoparietal control network; WMH, white matter hyperintensities

Finally, the results of mediation analyses showed that the FC slightly but significantly mediated the association of CRI‐education with visual‐spatial ability in the WMH without CI group (indirect effect: 0.006; 95% CI: 0.001, 0.013) (Figure 3A) and the association of CRI‐education with executive function in the WMH with CI group (indirect effect: −0.004; 95% CI: 0.003, 0.102) (Figure 3B).

#### 3.3.3 Association of each aspect of CR with the left FPCN

Like the findings in the right FPCN, CRI‐working activity and CRI‐leisure time activity were significantly associated with the left FPCN FC in the WMH without CI group (Figure S3A–C in Supplemental Material). In addition, the CRI‐working activity was associated with the left FPCN in the WMH with CI group (Figure S3B–D in Supplemental Material). Notably, CRI‐education was significantly associated with the FC in the right DLPFC in both WMH groups (Figure S4A–B in the Supplemental Material). However, the association was significantly positive and negative in the WMH without CI group (Figure S4C in Supplemental Material, *r* = 0.456, *p* < 0.001) and the WMH with CI group (Figure S4D in Supplemental Material, *r* = −0.430, *p* < 0.001), respectively. Similarly, the FPCN FC was positively and negatively associated with cognitive function in the WMH without CI group and the WMH with CI group, respectively (Figure S5 in Supplemental Material). No mediating effect was shown for the left FPCN.

## 4 DISCUSSION

The major findings of the present study are: (1) education has a positive effect on cognitive function in WMH subjects with or without CI, whereas the working activity and the leisure time activity have positive effects only in WMH subjects without CI. (2) FPCN FC in the DLPFC mediates the association between education and cognitive function in WMH subjects. These findings extend our understanding of the neural network mechanisms associated with CR and cognitive function in individuals with WMH.

This study investigated the role of each CR aspect in WMH subjects with or without CI. Although the three aspects of CR were significantly and positively correlated, they differed in their effects on cognitive function and the FPCN. A meta‐analysis of 135 studies explored the associations between CR and cognitive function in older adults and found that all three aspects of CR had positive associations with cognitive performance in almost all cognitive domains.^36^ A positive association between education and cognitive function was also shown in older adults with WMH.^37^ Our findings further showed that positive associations existed in WMH subjects with and without CI. In contrast, working activity and leisure time activity were only positively associated with cognitive function in those without CI. Therefore, education may contribute more to cognitive function in WMH subjects than working activity and leisure time activity. Higher educational attainment provides individuals with more knowledge, skills, and cognitive stimulation, thus improving cognitive performance.^38^ Higher educational attainment also has other benefits, including healthier lifestyles, more challenging jobs, better healthcare, and better controls for vascular risk factors.^39^, ^40^ These benefits may directly or indirectly affect cognitive function. It should be noted that although lower leisure time activity was shown in the WMH with CI group than in the other two groups, no significant effect of leisure time activity on cognitive function or the FPCN was found in WMH subjects with CI. The lower leisure time activity could reflect the functional impairment related to CI.

Another main finding of the present study was that the effects of CR on the FPCN were similar to those on cognitive function in WMH subjects. Several previous studies focusing on the associations between CR and FC or activities of functional networks showed that the associations were dependent on the cognitive status or disease stages.^19^, ^41^, ^42^, ^43^, ^44^ Interestingly, the present study only found cognitive status‐dependent effects of working activity and leisure time activity. Education had positive effects on the FPCN in WMH subjects regardless of their cognitive status, whereas working activity and leisure time activity mainly showed effects in those without CI. Furthermore, although the FC in the DLPFC partially mediated the associations between education and cognitive function in WMH subjects, the mediating patterns were the opposite. Education may maintain cognitive function by increasing the bilateral DLPFC FC in WMH subjects without CI but decreasing the FC in those with CI.

Previous studies investigating the relationship between the increased FC in the frontal lobe and cognitive function have yielded conflicting results. Some studies showed that increased frontal FC was associated with better cognitive performance in cognitively normal older adults, suggesting a compensatory neural process.^45^, ^46^, ^47^ According to the famous model named the "scaffolding theory of aging and cognition (STAC)", with the neuronal declines, compensatory scaffolding, i.e., compensatory recruitment or reallocation of cognitive resources, could be induced to maintain cognitive function and life‐course factors (including CR) could regulate the process.^48^, ^49^ However, other studies showed that increased frontal FC was associated with worse cognitive performance in healthy elderly or subjects with mild cognitive impairment,^50^, ^51^ suggesting that the increased FC might reflect pathology‐ or age‐related dedifferentiation of brain activities and could be harmful. In the present study, higher FC between bilateral DLPFC was associated with better cognitive performance in WMH subjects without CI and poorer cognitive performance in those with CI. The increased frontal FC suggests a compensatory process in subjects with WMH before the onset of CI but pathology‐related dedifferentiation of brain activities with the onset of CI.

Education usually represents early experiences that may not be easily improved in later life, while the other two aspects of CR, working activity and leisure time activity, are relatively more modifiable. Cognitive training can improve cognitive function in the general older population.^52^ Physical exercise, classified as a leisure time activity, also benefits specific cognitive domains, i.e., processing speed and attention, in older people with normal cognitive function.^53^ For subjects with cerebral small vessel disease, the beneficial effects of cognitive training and physical exercise exhibited at predementia stages.^54^, ^55^ Together with the cognitive status‐dependent effects of working activity and leisure time activity in this study, these findings suggested that improving CR at early stages could better preserve cognitive function.

Our study had some limitations. First, the present findings were based on cross‐sectional data and did not reflect the causal effects of CR on cognitive function and brain networks across stages of WMH. Longitudinal data would help investigate the causal effects of CR during the progression of WMH. Second, the recruitment diagnosis for WMH subjects with CI was based on clinical criteria. WMH might not have caused the CI in some participants, e.g., AD pathology‐related CI. Finally, the present study did not assess some vascular risk factors, e.g., hypertension, diabetes, atrial fibrillation, and related pharmacological treatments. These factors and treatments are associated with cognitive performance^56^, ^57^, ^58^ and may affect the present findings. Future studies should assess the effects of these factors on the relationship between WMH and cognitive function.

In conclusion, education showed a positive effect on cognitive function in WMH subjects with or without CI, whereas working activity and leisure time activity mainly showed positive effects in those without CI. The FC of the FPCN with the DLPFC mediated the effect of education on cognitive function in WMH subjects. These findings provide insights into the role of each CR proxy in maintaining the cognitive function of individuals with WMH and suggest the importance of improving CR before the onset of CI.

## CONFLICT OF INTEREST

The authors declare no conflict of interest.

## AUTHOR CONTRIBUTIONS

Yun Xu contributed to the conceptualization, project administration, methodology, and writing‐review & editing. Qing Ye contributed to the data analysis, formal analysis, methodology, and writing‐original draft. Huahong Zhu and Huiping Chen contributed to the methodology, data collection, data analysis, and writing‐original draft. Renyuan Liu, Lili Huang, Haifeng Chen, Yue Cheng, Ruomeng Qin, Pengfei Shao, Hengheng Xu, and Junyi Ma contributed to data collection. All authors reviewed and approved the manuscript.

## PATIENT CONSENT

All subjects provided written informed consent.

## Supporting information

Supplementary Material

## Data Availability

Deidentified data are available from the corresponding author upon reasonable request following the publication of this article.
