# Supplementary material for: Effects of cognitive reserve proxies on cognitive function and frontoparietal control network in subjects with white matter hyperintensities: A cross‐sectional functional magnetic resonance imaging study
Source: CNS Neurosci Ther. 2022 Mar 11;28(6):932–41. doi: 10.1111/cns.13824 (PMC9062549; doi:10.1111/cns.13824)
Supplement: Supplementary file 1 — Supplementary Material [file CNS-28-932-s001.docx]

**Supplementary Material**

**METHODS**

**Participants**

The study excluded individuals with neurodegenerative diseases (*e.g.*, Alzheimer's disease, Parkinson's disease, and Lewy body dementia), leukoencephalopathy of presumed nonvascular origin (*e.g.*, multiple sclerosis, neuromyelitis optica and radiation encephalopathy), history of ischemic stroke (diameter of infarct > 15 mm) or cardiogenic cerebral infarction, severe neurological diseases (*e.g.*, intracranial hemorrhage, traumatic brain injury, brain tumor and psychiatric disease), or severe systemic diseases (*e.g.*, cancer, anemia, thyroid dysfunction, and systemic lupus erythematosus).

**Assessment of cognitive function and cognitive reserve**

Memory function was measured using the Auditory-Verbal Learning Test-delayed recall and Rey-Osterrieth Complex Figure Test (CFT) with its 20-min Delayed Recall. Executive function was measured with the Trail Making Test B and Stroop Color and Word Test C. Processing speed was measured using the Trail Making Test A and Stroop Color and Word Test A. Visual-spatial ability was measured by the CFT with its immediate recall. Raw scores of each neuropsychological test (except the MMSE and MoCA) were z-transformed according to the following equation:

$$Z_{i}=\frac{(r_{i}-m)}{S}$$

$Z_{i}$ indicates the Z scores for the *i*th subject, $r_{i}$indicates the raw score for the *i*th subject, *m* indicates the average score for each test for each group, and *S* indicates the standard deviation of the test scores for each group. The individual scores for each domain were obtained by averaging the Z scores of the relevant neuropsychological tests.

To quantify CR, we conducted a survey with the CRIq and generated the composite index of CR, *i.e.*, the Cognitive Reserve Index (CRI). All participants with normal cognitive function were asked questions about three indicators of CR: education (years of education and possible training courses), working time activity (the levels of working activity, including unskilled manual work, skilled manual work, skilled nonmanual or technical work, professional occupation and highly intellectual occupation, and the number of years in each occupation over the lifespan) and leisure time activity (the frequency of the cognitively stimulating activities during leisure time). For those with CI, the questions were asked of a family caregiver who was familiar with the present and past habits of the subject. After collecting the self-reported information about CR, we obtained the scores for each of the three aspects of the CRIq: CRI-education, CRI-working activity, and CRI-leisure time activity, as well as the total CRI for each subject. The CRIq questionnaire and the Excel file for the automatic calculation of CRI scores are available at http://cri.psy.unipd.it.

**Magnetic resonance imaging acquisition**

All subjects underwent MRI scanning on a 3.0-Tesla MR scanner (Ingenia 3.0T, Philips Medical Systems, Eindhoven, Netherlands) with a 32-channel head coil. They were instructed to relax with eyes closed and stay awake during the scanning. The examination protocol included an resting-state fMRI sequence [repetition time (TR) = 2000 ms, echo time (TE) = 30 ms, flip angle (FA) = 90°, acquisition matrix = 64 × 64 , field of view (FOV) = 192 × 192 mm, thickness = 4.0 mm, gap = 0 mm, and number of slices = 35], a high-resolution three-dimensional (3D) T1-weighted sequence [TR = 9.8 ms, TE = 4.6 ms, FA = 8°,acquisition matrix = 256 × 256, FOV = 256 × 256 mm, thickness = 1.0 mm, gap = 0 mm, and number of slices = 192] and a T2-FLAIR sequence [TR = 4500 ms, TE = 344 ms, FA = 90°, acquisition matrix = 272 × 272, thickness =1.0 mm, gap = 0 mm, and number of slices = 200].

**Volume assessment of grey matter, whole brain and WMH**

Structural processing was performed using the VBM8 toolbox (http://dbm.neuro.uni–jena.de/vbm8) for Statistical Parametric Mapping software (SPM12, http://www.fil.ion.ucl.ac.uk/spm). First, the T1 images were segmented into grey matter (GM), white matter (WM), and cerebrospinal fluid (CSF). The segmented images were then normalized to the Montreal Neurological Institute (MNI) brain MRI template using affine and nonlinear spatial normalization and re-sampled to a voxel size of 1.5 × 1.5 × 1.5 mm. Jacobian modulation was applied to the segmented images, which was incorporated to compensate for the effect of spatial normalization. The extracted GM, WM, and CSF sets were then smoothed with an 8-mm full width at half maximum Gaussian filter to decrease the effects of individual variation in gyral anatomy and to increase the signal-to-noise ratio.

The volume of WMH lesions was evaluated on T1 and T2-FLAIR images using the LST toolbox version 2.0.151 (www.statistical-modelling.de/lst.html) for SPM12. Based on the lesion growth algorithm, the T1-weighted images were segmented into regions of GM, WM and CSF, and this information was combined with corresponding T2-FLAIR intensities to compute lesion belief maps. After setting a pre-chosen initial threshold (κ = 0.15) determined by 3 experienced radiologists to these maps, we obtained the initial binary lesion maps, which were subsequently grown along voxels that appear hyperintense on the T2-FLAIR images. The individual WMH volume was presented in a HTML report.

**FMRI preprocessing and network mapping**

The first ten volumes of each subject were discarded due to T1 equilibration effects. The slice time correction and head motion correction were performed owing to the acquisition-time differences between the slices within one volume and the motion effects during scanning. The head motion parameters indicated that no head motion exceeded 2 mm in x, y, or z translation or 2° in rotation. The resulting images were spatially normalized to MNI space with the mean echo-planar image (EPI) as the source volume, resampled to 3×3×3 mm^3^ voxels and smoothed with a Gaussian kernel of 6×6×6 mm. Then confounding covariates including WM, CSF and whole brain signals and Friston-24 head motion parameters were removed by multiple linear regression analysis. Finally, the band-pass filtering (0.01 - 0.1 Hz) was applied to the resulting data and the linear trend of time courses was removed.

Six-millimeter radius spheres centered at the bilateral dorsolateral prefrontal cortex (DLPFC) (MNI space: −42, 34, 20/44, 36, 20) served as seed regions for the bilateral FPCN. For each subject, a mean time series of the DLPFC was extracted as the reference time course for the FPCN. Next, *P*earson cross-correlation analysis was applied between the reference time course and time course of each voxel in the whole brain, and Fisher's Z-transformation was conducted to improve the normality of the correlation coefficients.

$$Z=0.5\times ln\frac{1+r}{1-r}$$

Eventually, the individual FC maps of the bilateral FPCN were obtained. The spatial maps were constructed by a one sample t test with the Resting-State fMRI Data Analysis Toolkit (REST 1.8, http://www.restfmri.net), and the threshold was set at a corrected *P* < 0.001, determined by Monte Carlo simulation for multiple comparisons (voxelwise *P* < 0.001, cluster size > 432 mm^3^). The binary images were used as the maps for the subsequent intra-FPCN analyses.

**FIGURES AND LEGENDS**

**
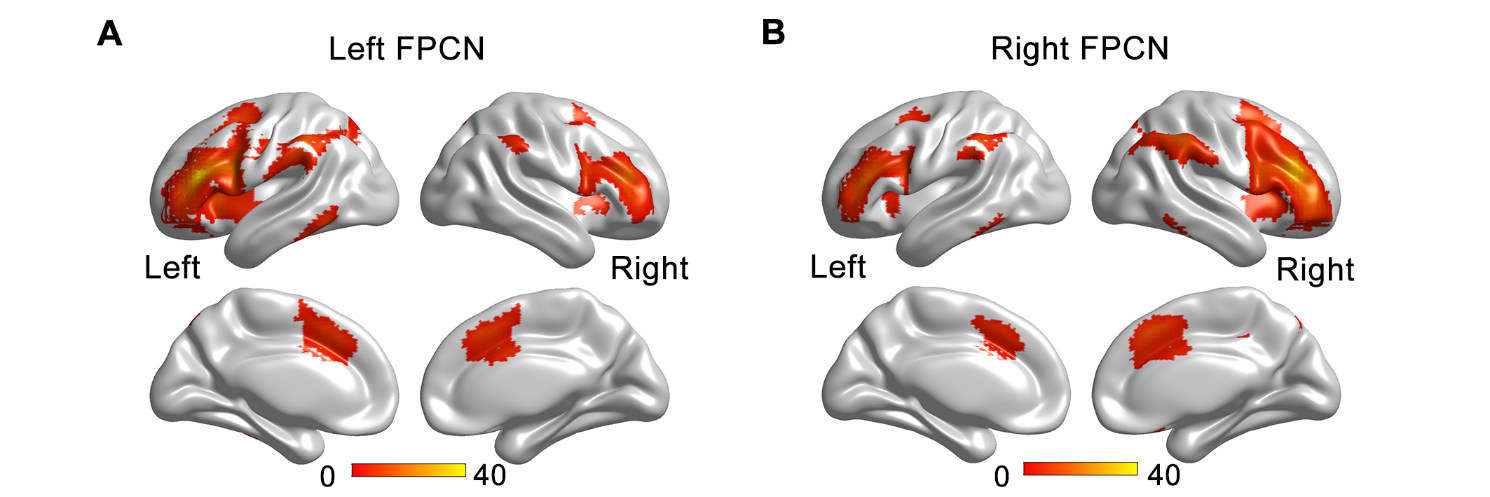
**

**Figure s1** The spatial maps of the bilateral FPCN. The thresholds were set at a corrected *P* < 0.001, determined by Monte Carlo simulation for multiple comparisons (voxel wise *P* < 0.001, cluster size > 432 mm^3^). The color bars present with *t* values. FPCN, frontoparietal control network.


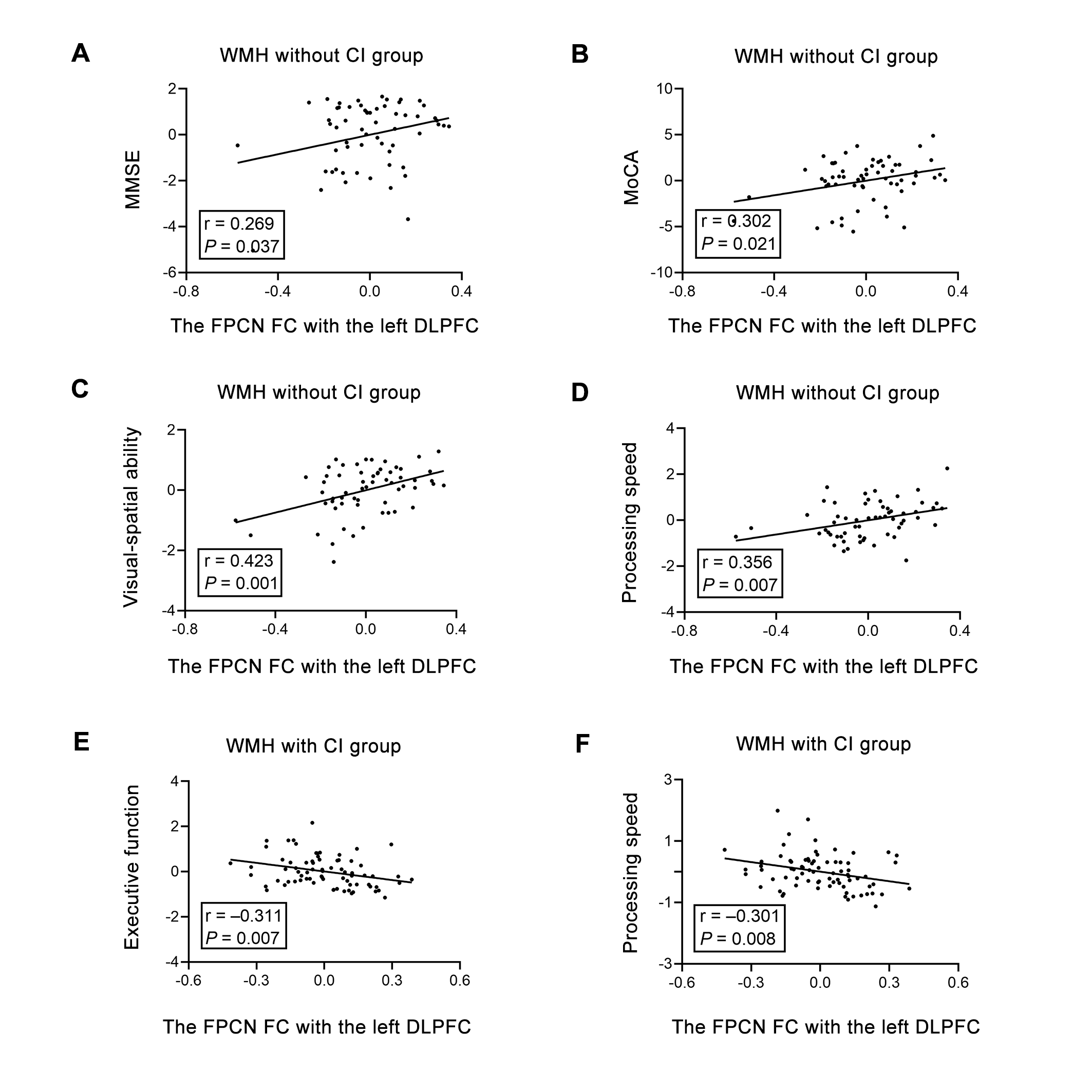


**Figure s2** The associations between cognitive function and the right FPCN FC with the left DLPFC. **(A-D)** In the WMH group without CI, FC was positively associated with the scores of MMSE and MoCA scores, visual-spatial ability, and processing speed. **(E)** and **(F)** In the WMH group with CI, FC was negatively associated with the scores of executive function and processing speed. The FC values were transformed to Z scores using Fisher's Z-transformation. CI, cognitive impairment; DLPFC, dorsolateral prefrontal cortex; FC, functional connectivity; FPCN, frontoparietal control network; MMSE, Mini Mental State Examination; MoCA, Montreal Cognitive Assessment; WMH, white matter hyperintensities.


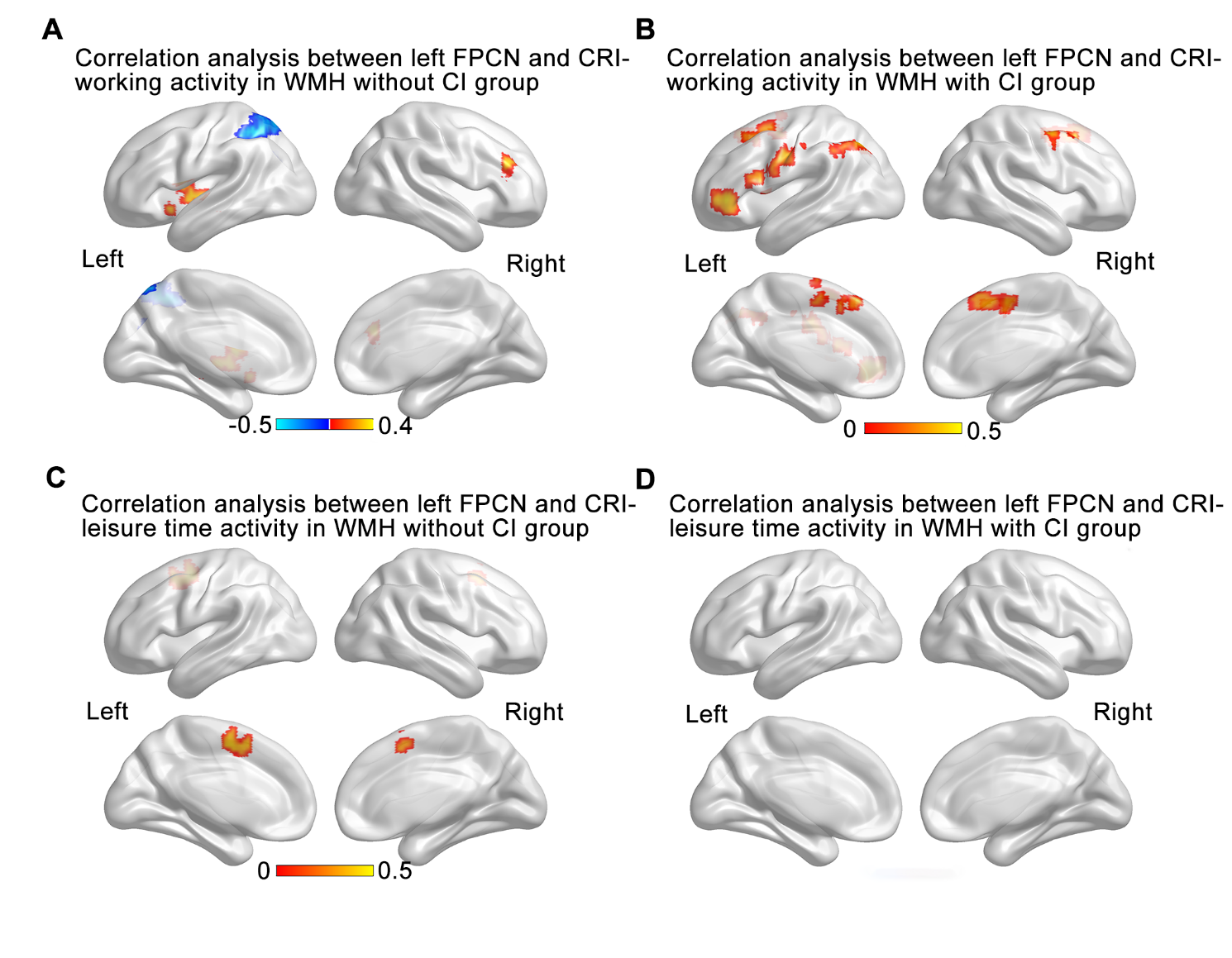


**Figure s3** The associations of CRI-working activity/CRI-leisure time activity with the left FPCN in the WMH groups. **(A)** and **(B)** CRI-working activity was significantly associated with FPCN FC in the frontal, parietal, and cingulate regions in the 2 WMH groups. **(C)** and **(D)** CRI-leisure time activity was significantly associated with FPCN FC in the cingulate regions in the WMH group without CI but not in the WMH group with CI. Correlation analyses were performed between the FPCN and each CRI aspect while controlling for age, gender, and grey matter images. The thresholds were set at a corrected *P* < 0.05, determined by Monte Carlo simulation for multiple comparisons (voxel-wise *P* < 0.05, cluster size > 2646 mm^3^). The color bars presented correlation coefficient. CI, cognitive impairment; CRI, cognitive reserve index; FC, functional connectivity; FPCN, frontoparietal control network; WMH, white matter hyperintensities.


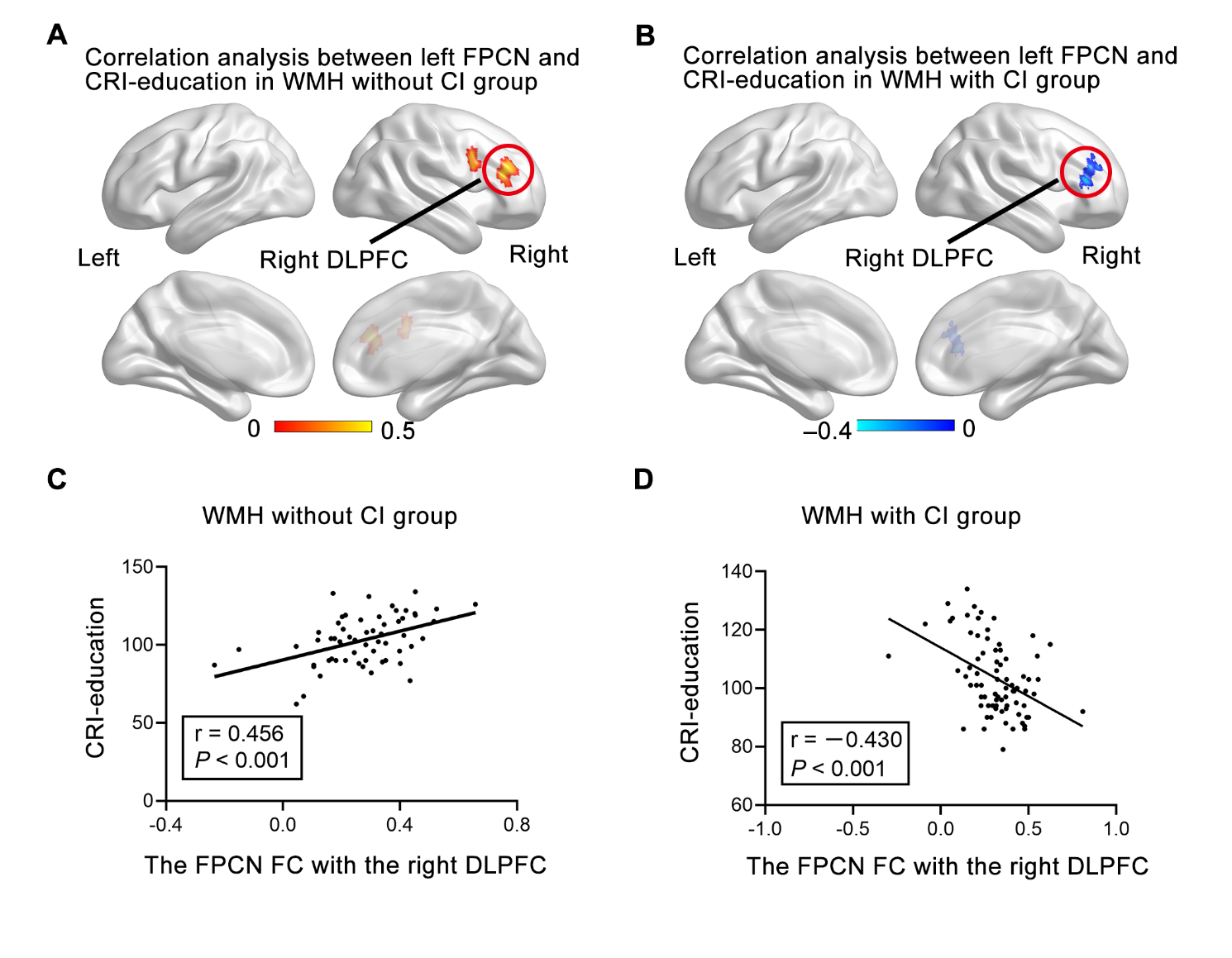


**Figure s4** The associations of CRI-education with the left FPCN in the 2 WMH groups. **(A)** and **(B)** CRI-education was significantly associated with FPCN FC in the right DLPFC in both WMH groups. Correlation analyses were performed between FPCN and each CRI aspect while controlling for age, gender, and grey matter images. The thresholds were set at a corrected *P* < 0.05, determined by Monte Carlo simulation for multiple comparisons (voxel-wise *P* < 0.05, cluster size > 2646 mm^3^). The color bars present correlation coefficients. **(C)** FPCN FC in the right DLPFC was positively associated with CRI-education in the WMH group without CI. **(D)** FPCN FC in the right DLPFC was negatively associated with CRI-education in the WMH group with CI. The FC values were transformed to Z scores using Fisher's Z-transformation. CI, cognitive impairment; CRI, cognitive reserve index; DLPFC, dorsolateral prefrontal cortex; FC, functional connectivity; FPCN, frontoparietal control network; WMH, white matter hyperintensities.


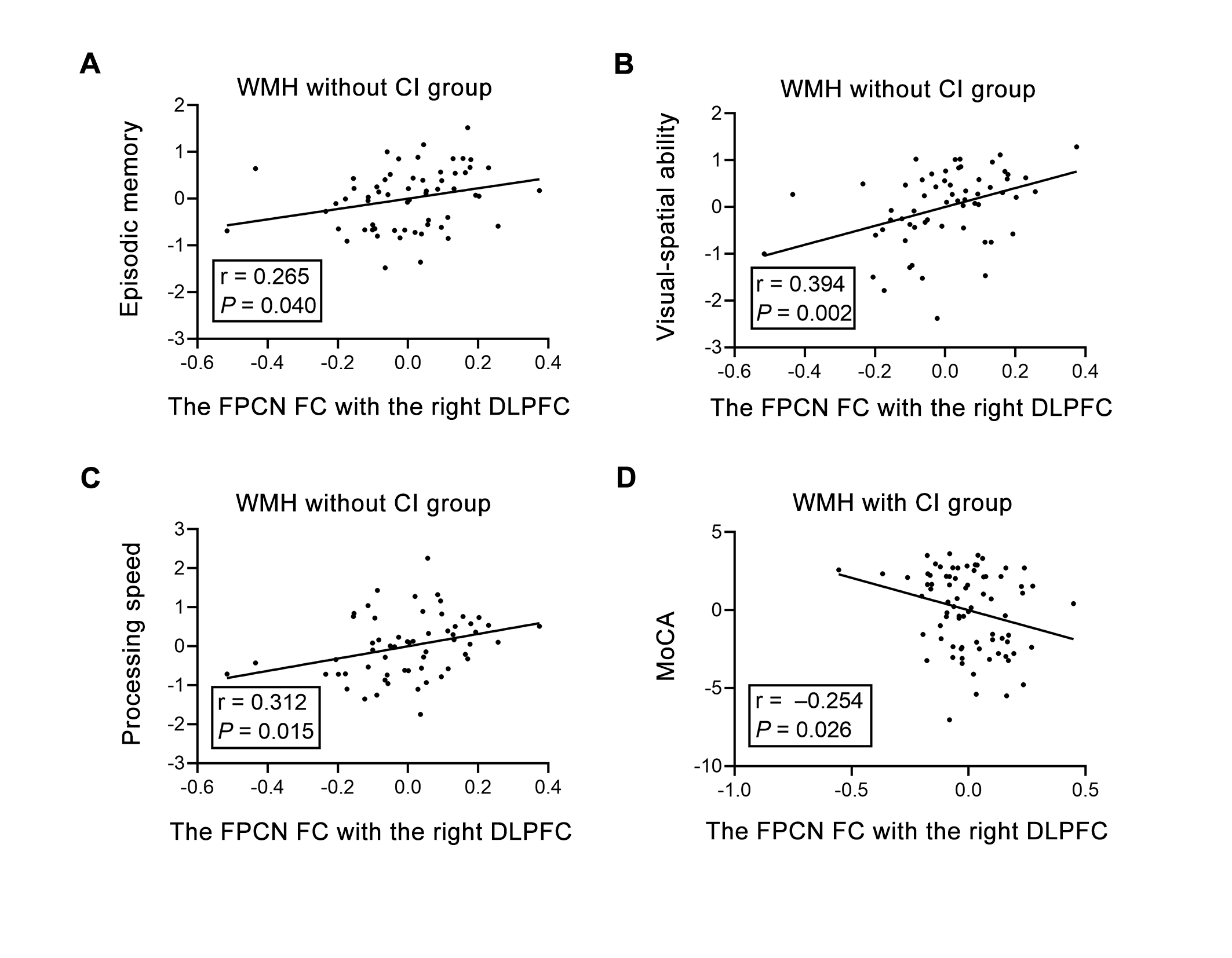


**Figure s5** The associations between cognitive function and the left FPCN FC with the right DLPFC. **(A-C)** In the WMH group without CI, FC was positively associated with episodic memory, visual-spatial ability, and processing speed. **(D)** In the WMH group with CI, FC was negatively associated with the MoCA scores. The FC values were transformed to Z scores using Fisher's Z-transformation. CI, cognitive impairment; DLPFC, dorsolateral prefrontal cortex; FC, functional connectivity; FPCN, frontoparietal control network; MoCA, Montreal Cognitive Assessment; WMH, white matter hyperintensities.

**TABLES**

| **Table s1 Associations among the three aspects of cognitive reserve** | | | | | | |  |
| --- | --- | --- | --- | --- | --- | --- | --- |
|  |  |  |  |  |  |  |  |
| **Groups**  **Items** | **HC** | | **WMH without CI** | | **WMH with CI** | |  |
|  | r | *P* | r | *P* | r | *P* |  |
| Education and working activity | 0.679 | 0.001^a^ | 0.626 | < 0.001^a^ | 0.296 | 0.009 |  |
| Education and leisure time activity | 0.340 | 0.001^a^ | 0.410 | 0.001^a^ | −0.018 | 0.877 |  |
| Working activity and leisure time activity | 0.290 | 0.004^a^ | 0.505 | < 0.001^a^ | 0.253 | 0.027 |  |
| Values depicted are correlation coefficients (r) and P values derived from Pearson's correlation analyses. ^a^*P* < 0.006 (0.05/9 tests) corrected with the Bonferroni principle. Abbreviations: CI, cognitive impairment; HC, healthy control; WMH, white matter hyperintensities. | | | | | | |  |
|  |  |  |  |  |  |  |  |
